# Are clinical trials dealing with severe infection fitting routine practices? Insights from a large registry

**DOI:** 10.1186/cc12734

**Published:** 2013-05-24

**Authors:** Yann-Erick Claessens, Philippe Aegerter, Hamdi Boubaker, Bertrand Guidet, Alain Cariou

**Affiliations:** 1 Department of Emergency Medicine, Centre Hospitalier Princesse Grace, MC-98012, Principalty of Monaco; 2 Department of Emergency Medicine, Hôpital Cochin, AP-HP, 27 rue du Faubourg Saint-Jacques, F-75679 Paris Cedex 14, France; 3 Faculté de médecine, Université Paris Descartes, F-75006 Paris, France; 4 Department of Public Health, Hôpital Ambroise Paré, AP-HP, 92104 Boulogne, France; 5 Université Versailles St-Quentin, UPRES EA 2506, Paris, France; 6 Department of Emergency Medicine, Centre Hospitalier Universitaire Fatouma Bourguiba, T-5019 Monastir, Tunisia; 7 Department of Intensive Care Medicine, Hôpital Saint-Antoine, AP-HP, F-75012 Paris, France; 8 Unité de Recherche en Épidémiologie Systèmes d'Information et Modélisation (U707), UPMC Université Paris 06 Inserm, F-75012 Paris, France; 9 Department of Intensive Care Medicine, Hôpital Cochin, AP-HP, F-75679 Paris Cedex 14, France

**Keywords:** sepsis, septic shock, randomized controlled trial, exclusion criteria, co-morbidity, cohort

## Abstract

**Introduction:**

Guidelines dealing with severe sepsis and septic shock mostly rely on randomized controlled trials (RCTs) to ensure the best standards of care for patients. However, patients included in high-quality studies may differ from the routine population and alter external validity of recommendations. We aimed to determine to what extent non-inclusion criteria of RCTs dealing with severe sepsis and septic shock may affect application of their conclusions in routine care.

**Methods:**

In a first step, the MEDLINE database was searched for RCTs treating severe sepsis and septic shock patients between 1992 and 2008, and non-inclusion criteria for these studies were abstracted. Two reviewers independently evaluated the articles, which were checked by a third reviewer. We extracted data on the study design, main intervention, primary endpoint, criteria for inclusion, and criteria for non-inclusion. In a second step, the distribution of the non-inclusion criteria was observed in a prospective multicenter cohort of severe sepsis and septic shock patients (Cub-Rea network, 1992 to 2008).

**Results:**

We identified 96 articles out of 7,012 citations that met the screening criteria. Congestive heart failure (35%) and cancer (30%) were frequent exclusion criteria in selected studies, as well as other frequent disorders such as gastrointestinal and liver diseases and all causes of immune suppression. Of the 67,717 patients with severe sepsis and septic shock in the Cub-Rea database, 40,325 (60%) experienced at least one of the main exclusion criteria, including 11% of congestive heart failure patients and 11% of cancer patients. In addition, we observed a significant trend for increasing number of patients with these criteria along time.

**Conclusion:**

Current exclusion criteria for RCTs dealing with severe sepsis and septic shock excluded most patients encountered in daily practice and limit external validity of the results of high-quality studies.

## Introduction

Since the early 1990s, considerable efforts have been provided to improve treatment and global management of patients suffering from severe infection. In August 1991, the American College of Chest Physicians and the Society of Critical Care Medicine released a landmark Consensus Conference agreeing on definitions to be applied to patients with severe infections [1]. Classifications were provided to assist clinicians and researchers including homogeneous and comparable populations in trials dealing with severe sepsis and septic shock. Following this conference, a number of major studies have been published that substantially modified the management and course of severe sepsis and septic shock patients. For the same period, the mortality of patients with severe infections did not decrease [2] or remained high, suggesting that physicians might be unaware of the scientific advances in the field of severe infection.

Severe sepsis is a major problem in intensive care, accounting for over 10 to 20% of stays of increased duration [3] and with a recent increase in hospitalization [4]. The most severe presentation is septic shock with an individual mortality that has recently slightly decreased [5] but with an overall specific mortality that has increased [6] due to the higher number of patients, in particular older patients [7].

This observation prompted scientific societies to develop a partnership that intended to provide standards of care for management of severe sepsis and septic shock, based on the highest levels of evidence published scientific knowledge. Key recommendations overviewed management of septic patients, including specific treatments such as antimicrobial therapy, hemodynamic management and use of adjunctive therapies, and supportive care such as sedation, tight glucose control and mechanical ventilation [8].

Implementation of these guidelines, however, has been offset by a number of barriers. Heterogeneous levels of equipment and resuscitation skills and overburden in ICUs compromise the quality of care delivered to septic patients, especially those requiring a high level of technical support [9,10]. Besides these organizational limitations, the way these guidelines are applicable or not to routine care patients is also controversial. One can suppose that differences between groups of homogeneous patients included in pivotal trials and real-life patients routinely admitted to the ICU may prevent guideline generalization. Even if non-inclusion criteria are known to explain these differences, their identification and frequency among routine ICU patients is lacking. As a consequence, it is actually difficult to assess the proportion of patients in whom the most current recommended treatments were in fact not tested. To specifically address whether international recommendations to treat septic patients fit routine ICU patients or not, we first checked for non-inclusion criteria in published randomized controlled trials (RCTs) dealing with severe sepsis and septic shock since 1992. In a second step, we investigated the frequency of these non-inclusion criteria in a large ICU population by using a multicenter ICU registry.

## Patients and methods

### First step: exclusion criteria in RCTs dealing with severe sepsis and septic shock

We first identify the most frequent non-inclusion criteria in RCTs dealing with severe infections.

#### Literature search strategy and inclusion criteria

The comprehensive literature searches included all trials published in PubMed from 1 January 1992 to 31 December 2008. This period corresponded to that from release of the first American College of Chest Physicians guidelines to the last update of bundles from the Surviving Sepsis Campaign [3].

We included all trials (positive and negative) that labeled the following MeSH keywords in the title and abstract: sepsis; severe sepsis; septic shock; treatment; randomized. We excluded trials in which no therapeutic intervention was undertaken, and trials on ARDS since sepsis is only one of the combining factors. Finally, we selected interventional trials in populations with severe sepsis and septic shock. All authors evaluated the eligibility of the trials, resolving disagreements by discussion and consensus.

#### Data extraction

Two authors (HB, Y-EC) independently extracted data that were checked by one author (AC). We noted the study period, year of publication, and geographic area where the study was performed. We detailed the study design: monocenter or multicenter, randomized or not, controlled or not, blind or open, financial disclosure (supported or not by the pharmaceutical industry or healthcare societies). We checked whether Bone criteria [1] were used or not to include patients, and whether mortality was the main evaluation criteria. We described the number of patients screened and excluded when available. Interventions (treatment, strategy, procedure) were classified as follow: vasopressors; fluid loading/global hemodynamic-based strategy; steroids; antimicrobial agents; modulation of immunity; coagulation-targeted therapy; miscellaneous. We abstracted whether the results related to the primary endpoint were positive or not.

For each trial, we carefully detailed the non-inclusion criteria that we distributed between the following categories: pregnancy; age categories (<18 years, >75 years); presence of congestive heart failure; presence of cancer (excluding local skin cancer and cancer healed for at least 6 months); gastrointestinal and liver disorder; HIV infection; solid organ transplantation; treatment with steroids; coagulation disorders or treatment; burns; renal replacement therapy; overweight; neutropenia; cerebrovascular stroke; miscellaneous.

### Second step: study cohort from the Cub-Rea database

This second step aimed to assess the distribution of previously identified exclusion criteria in a large ICU population.

#### Selection of patients from the database

The Collège des Utilisateurs de Bases de données en Réanimation (Cub-Rea) database has been described elsewhere [5,11,12]. In brief, the Cub-Rea network, created in 1992 by the Société de Réanimation de Langue Française (Paris, France), is a record of admissions to 40 adult ICUs in 35 hospitals located in and around Paris. According to French regulations for ethical use of computerized data, the Cub-Rea project was approved by the Comité National Informatique et Liberté.

Data were extracted from admissions to all the 40 ICUs participating in the database. We selected patients included in the database from 1992 to 2008. As published previously, the Cub-Rea group currently utilizes the common definition for severe sepsis and septic shock [1,5]. Data were extracted for 'septicemia', 'sepsis', 'severe sepsis' (defined as both an infection and an organ failure) and 'septic shock', and for demographic characteristics, dates of ICU admission and discharge, category (medical, scheduled or unscheduled surgical) and type of admission (community, hospital ward, or institution), and immune status (immune deficiency included HIV infection, ongoing malignancy, radiation or chemotherapy, high dose or chronic use of corticosteroids, immune-suppressive drugs). Data were also extracted for 'site of infection' and 'type of microorganisms', and for 'interventions'. The ICU length of stay was calculated using the number of calendar days between admission and discharge. Hospital mortality rates were available from 1997 and readmission rates during the same hospital stay were available from 1999 for all units.

Finally, we assessed the presence or absence of previously identified non-inclusion criteria among each of these routine ICU patients.

### Analyses

Unpaired *t *tests and χ^2 ^statistics were used for comparisons of continuous and nominal variables, respectively. The changes from 1993 to 2008 for relevant variables were analyzed by analysis of variance with the contrasts method and by Pearson chi-squared analysis with the Cochran-Armitage trend test for continuous and nominal variables, respectively. *P *<0.05 was considered significant in all multivariate analyses. Analyses were performed with SAS statistical software (SAS Institute, Cary, NC, USA).

## Results

Exclusion criteria in RCTs dealing with severe sepsis and septic shock

**Figure 1:**
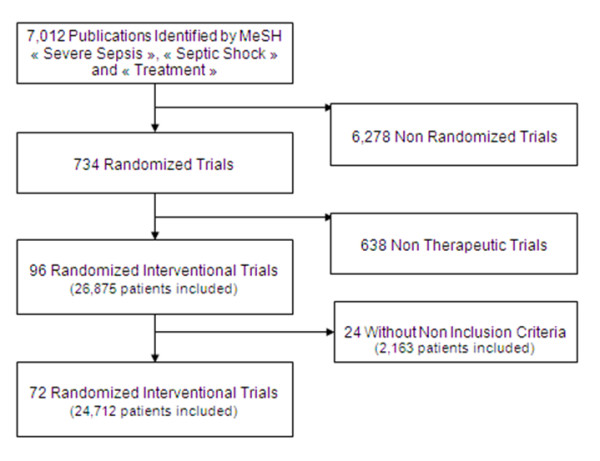
**Flow diagram of screened, eligible, and included randomized controlled trials, and number of patients included**.

A search using 'severe sepsis', 'septic shock' and 'treatment' as keywords in the PubMed database identified 7,012 publications from 1 January 1992 to 31 December 2008. Among these publications, 734 were 'randomized' trials. We selected 96 interventional trials including patients with severe sepsis and septic shock after careful assessment of each publication (Figure 1), corresponding to 26,875 included patients (see Additional file 1 for studies' characteristics and Additional file 2 for a complete publications' list). The main intervention referred to vasopressors in 38 trials; fluid loading/global hemodynamic-based strategy in eight trials; steroids in eight trials; modulation of immunity in 29 trials; coagulation-targeted therapy in 11 trials; and other adjunctive therapy in four trials. No study referred to antimicrobial agents. Results did not support the primary hypothesis in 63 studies. The number of patients screened and patients excluded were reported in only 23 trials.

Among the 96 selected studies, 24 (corresponding to 2,163 patients) did not mention any exclusion criteria. These trials were monocenter in 20 studies, and nine studies were controlled and eight studies were double blind. Results were positive for the primary endpoint in 33 studies. In these studies, the number of patients screened and excluded was not reported. Mortality was the primary endpoint in four studies.

The non-inclusion criteria used in the remaining 72 studies are reported in Table 1. Pregnant women were excluded from 46 studies, patients aged <18 years from 39 studies, and patients aged >75 years from four studies. The main underlying co-morbidities that led to patients' exclusion were congestive heart failure (34 studies) and cancer (30 studies). Gastrointestinal and liver disorders were also frequent non-inclusion criteria. In 33 studies, patients could not participate if they suffered from at least one cause of severe immune suppression (combining AIDS, neutropenia and transplant). Patients with coagulation disorders or treatment interfering with hemostasis could not be enrolled in 12 trials. Other miscellaneous non-inclusion criteria appeared in 60 trials.

**Table 1:** Exclusion criteria from randomized controlled trials dealing with severe sepsis and septic shock

Exclusion criteria	Number (%) of trials (*n *= 96)	Vasopressors (*n *= 37)	Fluid loading (*n *= 8)	Steroids (*n *= 8)	Modulation of immunity (*n *= 28)	Modulation of coagulation (*n *= 11)	Miscellaneous (*n *= 4)
Pregnancy	46 (47%)	16 (43%)	2 (25%)	4 (50%)	15 (54%)	6 (55%)	4 (100%)
Age <18 years	39 (40%)	7 (19%)	6 (75%)	5 (63%)	13 (46%)	7 (63%)	1 (25%)
Age >75 years	4 (4%)	1 (3%)	1 (13%)	1 (13%)	1 (4%)	0	0
Congestive heart failure	34 (35%)	13 (35%)	2 (25%)	3 (38%)	12 (43%)	2 (18%)	1 (25%)
Cancer	29 (30%)	7 (19%)	2 (25%)	3 (38%)	11 (39%)	6 (55%)	2 (50%)
Gastrointestinal and liver disorder	17 (18%)	6 (16%)	2 (25%)	2 (25%)	6 (21%)	3 (27%)	1 (25%)
Use of steroids	16 (16%)	0	0	7 (88%)	11 (39%)	0	0
HIV infection	15 (15%)	0	1 (13%)	3 (%)	9 (32%)	1 (9%)	0
Solid organ graft	14 (14%)	0	1 (13%)	4 (50%)	7 (25%)	2 (18%)	0
Coagulation abnormalities	12 (12%)	0	1 (13%)	2 (25%)	5 (18%)	6 (55%)	0
Burns	11 (11%)	0	1 (13%)	3 (38%)	4 (14%)	3 (27%)	0
Renal replacement therapy	11 (11%)	3 (8%)	1 (13%)	1 (13%)	3 (10%)	3 (27%)	0
Overweight	7 (7%)	0	0	0	2 (7%)	4 (36%)	0
Neutropenia	5 (5%)	1 (3%)	0	0	4 (14%)	0	0
Cerebrovascular stroke	3 (3%)	0	1 (13%)	0	0	1 (9%)	0

Results expressed as number (%). Exclusion criteria were not mutually exclusive.

### Study cohort from the Cub-Rea database

**Figure 2:**
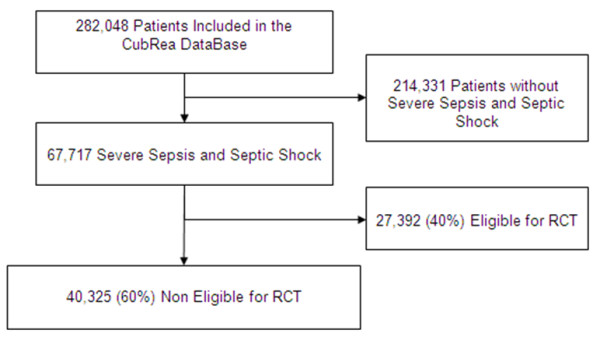
**Flow chart of patients from the Cub-Rea database (1992 to 2008)**. Cub-Rea, College des Utilisateurs de Bases de données en Réanimation; RCT, randomized controlled trial.

During the study period, 282,058 participants were admitted in the ICUs participating in the Cub-Rea network. Among these, 67,717 had severe sepsis and/or septic shock (Figure 2). Among the whole ICU population, the proportion of these patients with severe sepsis and/or septic shock progressively increased over time, and approximately doubled from 1992 (15.6%) to 2008 (30.9%, *P *<0.0001). At least one non-inclusion criterion identified in RCTs dedicated to severe infection was recorded in 40,325 (60%). Age >75 years was the most frequent non-inclusion criterion (Table 2), accounting for 23% of the population. Congestive heart failure and cancer were often present. Disorders leading to severe immune suppression were also frequently encountered. Of note, these exclusion criteria were frequently combined (Table 2). Other conditions leading to exclusion from RCTs were less frequent and each had an impact below 10% of the studied population. Interestingly, 14,009 (21%) patients had at least two main non-inclusion criteria.

**Table 2:** Exclusion criteria in patients from the Cub-Rea database with severe sepsis and septic shock.

Exclusion criteria	At least one criterion	One single criterion	Combination of two criteria	Combination of >2 criteria
	40,325	26,316	10,599	3,410
Age <18 years	377 (0.6%)	217 (0.8%)	84 (0.8%)	76 (2.2%)
Age >75 years	15,871 (23%)	9,733 (37%)	4,854 (46%)	1,284 (38%)
Pregnancy	80 (0.1%)	43 (0.1%)	28 (0.3%)	9 (0.3%)
Congestive heart failure	7,457 (11%)	2,848 (11%)	3,364 (32%)	1,245 (37%)
Cancer	7,266 (11%)	2,810 (11%)	2,820 (27%)	1,636 (45%)
Severe immune suppression^a^	8,930 (13%)	3,600 (14%)	2,975 (28%)	2,355 (69%)
AIDS	3,519 (5%)	2,400 (9%)	759 (7%)	360 (11%)
Neutropenia	2,949 (4%)	492 (2%)	1,270 (12%)	1,187 (35%)
Solid organ graft	2,462 (4%)	708 (3%)	946 (9%)	808 (24%)
Coagulation abnormalities	5,481 (8%)	1,567 (6%)	2,231 (22%)	1,613 (47%)
Gastrointestinal and liver disorders	5,416 (8%)	2,197 (8%)	2,003 (19%)	1,216 (36%)
Chronic renal failure requiring RTT	1,990 (3%)	660 (3%)	796 (8%)	534 (16%)
Overweight	2,027 (3%)	910 (3%)	716 (7%)	401 (12%)
Cerebrovascular stroke	1,953 (3%)	1,055 (4%)	639 (6%)	259 (8%)
Burns	160 (0.2%)	69 (0.2%)	55 (0.5%)	36 (0.1%)

Results expressed as number (%). CuB-Rea, College des Utilisateurs de Bases de données en Réanimation; RTT, renal replacement therapy. ^a^Severe immune suppression category combines patients with AIDS or neutropenia or transplant. Transplant category combines patients with bone marrow, kidney, liver, and heart or lung transplant.

**Figure 3:**
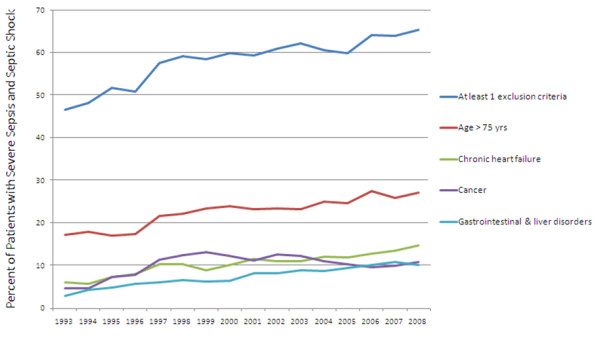
**Time trends of main exclusion criteria in septic patients from Cub-Rea database (1993 to 2008)**. Year 1992 was removed from the graph because of nonrepresentative values related to the small sample. Cub-Rea, College des Utilisateurs de Bases de données en Réanimation.

All along the study period, a time trend was observed that corresponds to an increasing number of patients with at least one non-inclusion criterion over years. A majority of patients were free from non-inclusion criteria in 1992, while 65.4% experienced at least one exclusion criterion in 2008. Patients >75 years of age represented <20% before 1997 and up to 27% in the late 2000s (Figure 3). This time-dependent change was observed for most non-inclusion criteria. Among these criteria, overweight was subject to the highest increase, as it was registered in <1% of the population until 1996, and reached 5.7% in 2008 (*P *<0.0001). The number of pregnant women did not change over time (*P *= 0.99). By contrast, AIDS (*P *<0.0001) (see Additional file 3), cerebrovascular stroke (*P *<0.0001) and burns (*P *= 0.02) significantly decreased during the study period.

Finally, we compared patients with and without non-inclusion criteria. Patients with at least one non-inclusion criterion were older, had higher Simplified Acute Physiological Score II, more frequently required vasopressors and renal replacement therapy, and had worse outcome (*P *<0.0001) (Table 3).

**Table 3:** Characteristics and eligibility/non-eligibility distribution of severe sepsis and septic shock patients from Cub-Rea

	Total population (*n *= 67,717)	Eligible for RCT (*n *= 27,392)	Non-eligible for RCT (*n *= 40,325)	*P *value
Age, years	64 (50 to 75)	58.2 (46 to 68)	69 (53 to 79)	<0.0001
Gender, male	42,977 (63%)	18,177 (66%)	24,800 (62%)	<0.0001
Charlson Index ≥2	8,366 (12%)	1,254 (5%)	7,112 (18%)	<0.0001
Referred from ED	28,847 (42%)	12,730 (46%)	16,117 (40%)	<0.0001
SAPS 2	46 (34 to 63)	38 (25 to 53)	49 (36 to 66)	<0.0001
Mechanical ventilation	52,783 (78%)	21,290 (78%)	31,493 (78%)	0.249
Renal replacement therapy	12,654 (19%)	3,429 (13%)	9,225 (23%)	<0.0001
Vasopressors	40,126 (59%)	14,211 (52%)	25,915 (64%)	<0.0001
ICU mortality	22,970 (34%)	6,613 (24%)	16,357 (41%)	<0.0001
In-hospital mortality	22,447 (33%)	6,088 (22%)	16,359 (41%)	<0.0001

Characteristics of patients from the College des Utilisateurs de Bases de données en Réanimation (Cub-Rea) database with severe sepsis and septic shock, and distribution according to their eligibility or non-eligibility to randomized controlled trials (RCTs). Results expressed as number (%) and median (interquartile range). *P *<0.05 was statistically significant. ED, emergency department; SAPS, Simplified Acute Physiological Score.

## Discussion

In this article we demonstrate that RCTs dedicated to treatment of severe sepsis and septic shock do not fit a large part of the patients that are currently admitted to ICUs with severe infection. Patients with common co-morbidities, usual medications, cancer and various immune deficiencies were not included in clinical trials dealing with severe sepsis and septic shock although such patients are likely to develop severe infections [13]. Whether the results of these trials are applicable to these frequently encountered ICU patients is questionable.

RCTs are believed to currently provide the best level of evidence that ensures efficacy of drugs interventions or strategies. Regarding severe infections, their results represent the basement of evidence-based clinical knowledge and practices as recommendations of the Surviving Sepsis Campaign [8]. Feasibility of such trials requires homogeneous populations with selective characteristics to allow group comparisons and internal validation. Characteristics of the population are therefore tightly selected but lead to exclusion of potential participants. For instance, it has been reported that 23% trials included in meta-analyses excluded no patients [14], and the results were more significant in studies that excluded patients. In the present study, the majority (75%) of RCTs devoted to severe sepsis and septic shock had non-inclusion criteria. Selecting patients is also believed to decrease uncontrolled and unknown adverse events in patients with severe underlying disorders. As a consequence, more fragile patients cannot enter most RCTs. Patients enrolled in clinical trials therefore sometimes differ from the actual target population. This may result in a paradox where methodology oversights clinical relevance. Research conducted by more than 300 analysts observed that older people were often excluded from studies focused on Alzheimer's dementia, arthritis and incontinence [15]. RCTs may therefore lack external validity. Consequently, efficacy and adverse outcome can be ignored when translating scientific evidence into daily practice.

Clinical practice addresses complex patients suffering from multiple health problems that require multiple medications. In a primary care population, patients eligible for five RCTs dealing with hypertension had 5 to 11 chronic conditions [16]. Ideally, RCTs would include more complex and severe patients to allow translation of results in daily practice. A recent review examined the exclusion criteria in 283 RCTs published in high-quality medical journals [17]. The subject of interest in these articles was infectious diseases in 55 (19%) studies and critical care in eight (2.8%). Exclusion related to medication and co-morbidities were frequent and poorly justified (for details, see Additional file 4). As an example, cardiac disorders are excluded from 24% of RCTs published in the selected set of journals [17]. In western countries, congestive heart failure is the most common underlying disorder and the leading cause of death [12]. Additionally congestive heart failure is a major co-morbidity associated with death in septic patients. In our study, patients with congestive heart failure were excluded in 34 (35%) articles whereas the disorder was encountered in 11% patients with severe infections from our database (Additional files 5 and 6).

Obviously disorders or treatments that deregulate innate and adaptative immunity increase the risk for severe infections. Neutropenia, steroids, cancer and especially hematological malignancies are basically responsible for a burden of infection. In a report of 283 trials including 35 RCTs for treatment of oncologic and hematological malignancies, cancer patients were excluded in 46 (16%) studies and those with blood disorders in 59 (21%) [17]. Here we show that these were frequent exclusion criteria for patients with severe infection, whereas they were frequently encountered at the bedside. Besides these exclusion criteria that can be forecast, other frequent conditions might be underperceived as exclusion criteria whereas they frequently impair the eligibility of septic patients. This particularly applies to gastrointestinal and liver disorders.

Pharmacological properties of medications are modified in physiological changes. Anticipating these variations is sometimes challenging. Obesity, an increasing condition in western countries, is illustrative for this paradigm. In obese patients, pharmacokinetics of drugs may change as the volume distribution differs from nonobese patients. We observed that overweight was a criterion for exclusion in 7% trials while 18 to 32% of American citizens present a body mass index >30 [18]. We observed in our population that obesity was a condition that increased over time whereas it did not reach the incidence recorded in North America. This may be related to a different distribution of this condition in our area [19] or to recent perception of the problem or inadequate evaluation and underestimation of overweight. As obese patients may be at risk to develop severe infections [20], and because their prognosis is worsened [19], people with overweight should enter RCTs dealing with severe sepsis and septic shock.

We observed that a minority of trials dealing with severe infections excluded older patients. The burden of infection encountered in western countries is related to ageing. Additionally, age and mortality are closely related in infectious diseases [21,22]. A number of reports recently warned physicians on the exclusion of older patients from RCTs [23,24]. More than 38% of RCTs excluded patients over the age of 65 [17]. In trials devoted to treatment of cancer, only 25% of patients were older than 65 [25]. In our study, older patients were excluded from only 4% of studies but the most common cutoff was 75 years, which can be responsible for an underestimating of age impact on non-inclusion.

The weight of co-morbidities on patients' prognosis is a usual concept in several fields of medicine. Their impact has been extensively described in infectious disorders [13,26,27]. In this setting, underlying disorders are likely to impair prognosis by decreasing host defense against the microorganism [28]. Alternatively, patients are unable to face increasing oxygen demand because of impaired physiological adaptation. In brief, co-morbidities expose the patient to more severe infections [28]. Whereas excluding patients because of co-morbidities allows recruitment of a homogeneous population in RCTs, it may be detrimental for translation of evidence into daily practice.

A major issue is to determine barriers to participate in a clinical trial. Refusal occurs in one out of five patients approached. Factors that influence the decision to decline the invitation to enter a trial are poorly known [29]. The most important reasons for failing entry into a study remain lack of an adequate trial and unmet inclusion criteria. This suggested a lack of pragmatic studies addressing frequent clinical questions in patients with common characteristics [30].

We identified several limitations in this study. First, there might be some differences between non-inclusion criteria extracted from RCTs and patients' characteristics. However, even if they exist, their relevance was limited and should have induced a negligible bias. Perhaps more important, we were unable to identify both coagulation disorders and use of steroids among the database population. Consequently, no firm conclusion can be drawn from our results regarding these two criteria. Also we did not restrict our analysis to positive studies but also to negative studies, since we considered that these negative studies could have influenced the sepsis guidelines in a similar manner. Finally, we tested only one French database population, and a replication in a different ICU population could have been useful. Whereas we provide data from a large multicenter series, patients we selected for severe sepsis and septic shock may differ from other cohorts, even in western countries. Indeed, assessment of these diagnoses may be subjective, and the incidence of underlying disorders may be not representative for other areas.

## Conclusion

Here we demonstrate that RCTs dealing with severe sepsis and septic shock infrequently include patients with conditions that usually coexist and predispose to severe infection in routine ICU patients. Moreover, we also show that the frequency of these non-inclusion conditions increased over recent years. Excluding patients from analysis of intervention trials presumably results in biased estimates of treatment effects, because patients excluded may correspond to the actual target population [14]. These findings advocate for extension of entry criteria for participants with multiple co-morbidities as they constitute the majority of patients with severe sepsis and septic shock.

## Key messages

• RCTs dealing with severe sepsis and septic shock support current guidelines; however, these studies use stringent exclusion criteria that lead to exclusion of numerous patients.

• In an important cohort of patients with severe sepsis and septic shock, 60% experienced at least one of the main exclusion criteria for RCTs dedicated to this disorder.

• A significant trend for increasing number of patients with these criteria is observed with time.

## Abbreviations

CuB-Rea: College des Utilisateurs de Bases de données en Réanimation; RCT: randomized controlled trial.

## Competing interests

The authors declare that they have no competing interests.

## Authors' contributions

Y-EC conceived the study, proceeded to selection of the studies used for the paper, and participated in data extraction and redaction of the manuscript. PA was responsible for extraction of data from the Cub-Rea database, statistical treatment of the data and redaction of the manuscript. HB proceeded to selection of the studies used for the paper and participated in data extraction and redaction of the manuscript. BG was responsible for the Cub-Rea network and database, and participated in conception of the study and critical review of the manuscript. AC participated in the study conception and design, and in redaction of the manuscript. All authors approved the final manuscript. Physicians from the Cub-Rea group participated in daily implementation of the database.

## Supplementary Material

Additional file 1 **a table presenting the characteristics of selected trials**. *Categories that were not mutually exclusive. Click here for file

Additional file 2 **a complete list of publications selected for the study**. Click here for file

Additional file 3 **a figure showing the time trends of severe immune suppression in septic patients from the Cub-Rea Database (1993 to 2008)**. Severe immune suppression combined transplant, neutropenia and AIDS. Year 1992 was removed from the graph because of nonrepresentative values related to the small sample. Click here for file

Additional file 4 **a list presenting the classification of non-inclusion criteria according to their justification **[17]. Click here for file

Additional file 5 **a table presenting the frequency of poorly justified reasons among main non-inclusion criteria across studies' categories **[17]. Results are expressed as number of poorly justified reason for non-inclusion/number of studies with each non-inclusion criterion (%). Click here for file

Additional file 6 **a table presenting the non-inclusion criteria in studies published in high impact factor journals**. Click here for file
