# Supplementary material for: Are clinical trials dealing with severe infection fitting routine practices? Insights from a large registry
Source: Crit Care. 2013 May 24;17(3):R89. doi: 10.1186/cc12734 (PMC3706971; doi:10.1186/cc12734)
Supplement: Additional file 1 — a table presenting the characteristics of selected trials. *Categories that were not mutually exclusive. [file cc12734-S1.DOC]

**Additional data**

Table. Characteristics of selected trials. * corresponds to categories that were not mutually exclusive.

| *Characteristics* | *No (%) of trials (n=96)* |
| --- | --- |
| Disorder  Severe sepsis  Septic shock  Severe sepsis and septic shock  Selection using Bone criteria  Yes  No  Year of publication  1992-1996  1997-2000  2001-2004  2005-2009  Funding  Industry  Other  None declared  No of participating centers  Single  Multiple  Controlled  Yes  No  Blind  Yes  No  Death reduction as the main criteria  Yes  No  No of patients entering the trial  <50  50-99  100-299  300-599  600-999  1000 and above  Geographic areas*  Western Europe  Eastern Europe  North America  South America  Oceania  Asia  Africa | 59 (21%)  25 (26%)  12 (30%)  60 (62%)  36 (38%)  20 (21%)  25 (26%)  29 (30%)  22 (23%)  40 (42%)  13 (14%)  33 (34%)  59 (61%)  37 (39%)  63 (65%)  33 (35%)  47 (49%)  49 (51%)  39 (41%)  57 (59%)  51 (53%)  11 (12%)  12 (13%)  6 (6%)  7 (7%)  8 (8%)  74 (76%)  2 (2%)  24 (25%)  2 (2%)  10 (11%)  2 (2%)  2 (2%) |
