# Supplementary material for: Are clinical trials dealing with severe infection fitting routine practices? Insights from a large registry
Source: Crit Care. 2013 May 24;17(3):R89. doi: 10.1186/cc12734 (PMC3706971; doi:10.1186/cc12734)
Supplement: Additional file 2 — a complete list of publications selected for the study. [file cc12734-S2.DOC]

**Additional file 2. Complete list of publications selected for the study.**

1. Abraham E, Glauser MP, Butler T, et al; [p55 Tumor necrosis factor receptor fusion protein in the treatment of patients with severe sepsis and septic shock. A randomized controlled multicenter trial. Ro 45-2081 Study Group.](http://www.ncbi.nlm.nih.gov/pubmed/9153367) *JAMA*. 1997;277:1531-1538.
2. Abraham E, Anzueto A, Gutierrez G, et al; [Double-blind randomised controlled trial of monoclonal antibody to human tumour necrosis factor in treatment of septic shock. NORASEPT II Study Group.](http://www.ncbi.nlm.nih.gov/pubmed/9734938) *Lancet*. 1998;351:929-933.
3. Abraham E, Laterre PF, Garbino J, et al; [Lenercept (p55 tumor necrosis factor receptor fusion protein) in severe sepsis and early septic shock: a randomized, double-blind, placebo-controlled, multicenter phase III trial with 1,342 patients.](http://www.ncbi.nlm.nih.gov/pubmed/11373411) *Crit Care Med*. 2001;29:503-510.
4. Abraham E, Reinhart K, Svoboda P, et al; [Assessment of the safety of recombinant tissue factor pathway inhibitor in patients with severe sepsis: a multicenter, randomized, placebo-controlled, single-blind, dose escalation study.](http://www.ncbi.nlm.nih.gov/pubmed/11700399) *Crit Care Med*. 2001;29:2081-2089.
5. Abraham E, Reinhart K, Opal S, et al; [Efficacy and safety of tifacogin (recombinant tissue factor pathway inhibitor) in severe sepsis: a randomized controlled trial.](http://www.ncbi.nlm.nih.gov/pubmed/12851279) *JAMA*. 2003;290:238-247.
6. Abraham E, Laterre PF, Garg R, et al; Administration of Drotrecogin Alfa (Activated) in Early Stage Severe Sepsis (ADDRESS) Study Group. [Drotrecogin alfa (activated) for adults with severe sepsis and a low risk of death.](http://www.ncbi.nlm.nih.gov/pubmed/16192478) *N Engl J Med*. 2005;353:1332-1341.
7. Albanèse J, Leone M, Delmas A, Martin C; [Terlipressin or norepinephrine in hyperdynamic septic shock: a prospective, randomized study.](http://www.ncbi.nlm.nih.gov/pubmed/16148457) *Crit Care Med*. 2005;33:1897-1902.
8. Alía I, Esteban A, Gordo F, et al; [A randomized and controlled trial of the effect of treatment aimed at maximizing oxygen delivery in patients with severe sepsis or septic shock.](http://www.ncbi.nlm.nih.gov/pubmed/10027447) *Chest*. 1999;115:453-461.
9. Angstwurm MW, Engelmann L, Zimmermann T, et al; [Selenium in Intensive Care (SIC): results of a prospective randomized, placebo-controlled, multiple-center study in patients with severe systemic inflammatory response syndrome, sepsis, and septic shock.](http://www.ncbi.nlm.nih.gov/pubmed/17095947) *Crit Care Med*. 2007;35:118-126.
10. Annane D, Sébille V, Charpentier C, et al; [Effect of treatment with low doses of hydrocortisone and fludrocortisone on mortality in patients with septic shock.](http://www.ncbi.nlm.nih.gov/pubmed/12186604) *JAMA*. 2002;288:862-871.
11. [Annane D](http://www.ncbi.nlm.nih.gov/pubmed?term="Annane D"%5BAuthor%5D), [Vignon P](http://www.ncbi.nlm.nih.gov/pubmed?term="Vignon P"%5BAuthor%5D), [Renault A](http://www.ncbi.nlm.nih.gov/pubmed?term="Renault A"%5BAuthor%5D), et al; Norepinephrine plus dobutamine versus epinephrine alone for management of septic shock: a randomised trial. *[Lancet.](http://www.ncbi.nlm.nih.gov/pubmed?term=annane d epinephrin lancet sepsis" \l "%23)* 2007;370:676-684.
12. [Avontuur JA](http://www.ncbi.nlm.nih.gov/pubmed?term="Avontuur JA"%5BAuthor%5D), [Tutein Nolthenius RP](http://www.ncbi.nlm.nih.gov/pubmed?term="Tutein Nolthenius RP"%5BAuthor%5D), [van Bodegom JW](http://www.ncbi.nlm.nih.gov/pubmed?term="van Bodegom JW"%5BAuthor%5D), [Bruining HA](http://www.ncbi.nlm.nih.gov/pubmed?term="Bruining HA"%5BAuthor%5D); Prolonged inhibition of nitric oxide synthesis in severe septic shock: a clinical study. *[Crit Care Med.](http://www.ncbi.nlm.nih.gov/pubmed/9559602#%23)* 1998;26:660-667.
13. Bakker J, Grover R, McLuckie A, et al; [Administration of the nitric oxide synthase inhibitor NG-methyl-L-arginine hydrochloride (546C88) by intravenous infusion for up to 72 hours can promote the resolution of shock in patients with severe sepsis: results of a randomized, double-blind, placebo-controlled multicenter study (study no. 144-002).](http://www.ncbi.nlm.nih.gov/pubmed/14707554) *Crit Care Med*. 2004;32:1-12.
14. Baudo F, Caimi TM, de Cataldo F, et al; [Antithrombin III (ATIII) replacement therapy in patients with sepsis and/or postsurgical complications: a controlled double-blind, randomized, multicenter study.](http://www.ncbi.nlm.nih.gov/pubmed/9609411) *Intens Care Med*. 1998;24:336-342.
15. Bernard GR, Ely EW, Wright TJ, et al; [Safety and dose relationship of recombinant human activated protein C for coagulopathy in severe sepsis.](http://www.ncbi.nlm.nih.gov/pubmed/11700394) *Crit Care Med*. 2001;29:2051-2059.
16. Bernard GR, Vincent JL, Laterre PF, et al; Recombinant human protein C Worldwide Evaluation in Severe Sepsis (PROWESS) study group. [Efficacy and safety of recombinant human activated protein C for severe sepsis.](http://www.ncbi.nlm.nih.gov/pubmed/11236773) *N Engl J Med*. 2001;344:699-709.
17. Berré J, De Backer D, Moraine JJ, Mélot C, Kahn RJ, Vincent JL ; [Dobutamine increases cerebral blood flow velocity and jugular bulb hemoglobin saturation in septic patients.](http://www.ncbi.nlm.nih.gov/pubmed/9118652) *Crit Care Med*. 1997;25:392-398.
18. [Bollaert PE](http://www.ncbi.nlm.nih.gov/pubmed?term="Bollaert PE"%5BAuthor%5D), [Charpentier C](http://www.ncbi.nlm.nih.gov/pubmed?term="Charpentier C"%5BAuthor%5D), [Levy B](http://www.ncbi.nlm.nih.gov/pubmed?term="Levy B"%5BAuthor%5D), [Debouverie M](http://www.ncbi.nlm.nih.gov/pubmed?term="Debouverie M"%5BAuthor%5D), [Audibert G](http://www.ncbi.nlm.nih.gov/pubmed?term="Audibert G"%5BAuthor%5D), [Larcan A](http://www.ncbi.nlm.nih.gov/pubmed?term="Larcan A"%5BAuthor%5D); Reversal of late septic shock with supraphysiologic doses of hydrocortisone. *[Crit Care Med.](http://www.ncbi.nlm.nih.gov/pubmed?term=bollaert  steroid sepsis 1998" \l "%23)* 1998;26:645-650.
19. Bone RC, Balk RA, Fein AM, et al; [A second large controlled clinical study of E5, a monoclonal antibody to endotoxin: results of a prospective, multicenter, randomized, controlled trial. The E5 Sepsis Study Group.](http://www.ncbi.nlm.nih.gov/pubmed/7774238) *Crit Care Med*. 1995;23:994-1006.
20. [Bourgoin A](http://www.ncbi.nlm.nih.gov/pubmed?term="Bourgoin A"%5BAuthor%5D), [Leone M](http://www.ncbi.nlm.nih.gov/pubmed?term="Leone M"%5BAuthor%5D), [Delmas A](http://www.ncbi.nlm.nih.gov/pubmed?term="Delmas A"%5BAuthor%5D), [Garnier F](http://www.ncbi.nlm.nih.gov/pubmed?term="Garnier F"%5BAuthor%5D), [Albanèse J](http://www.ncbi.nlm.nih.gov/pubmed?term="Albanèse J"%5BAuthor%5D), [Martin C](http://www.ncbi.nlm.nih.gov/pubmed?term="Martin C"%5BAuthor%5D); Increasing mean arterial pressure in patients with septic shock: effects on oxygen variables and renal function. *[Crit Care Med.](http://www.ncbi.nlm.nih.gov/pubmed?term=bourgoin norepinephrin 2005" \l "%23)* 2005;33:780-786.
21. Brunkhorst FM, Engel C, Bloos F, et al; [Intensive insulin therapy and pentastarch resuscitation in severe sepsis.](http://www.ncbi.nlm.nih.gov/pubmed/18184958)*N Engl J Med*. 2008;358:125-139.
22. Briegel J, Forst H, Haller M, et al; [Stress doses of hydrocortisone reverse hyperdynamic septic shock: a prospective, randomized, double-blind, single-center study.](http://www.ncbi.nlm.nih.gov/pubmed/10321661) *Crit Care Med*. 1999;27:723-732.
23. Busund R, Koukline V, Utrobin U, Nedashkovsky E; [Plasmapheresis in severe sepsis and septic shock: a prospective, randomised, controlled trial.](http://www.ncbi.nlm.nih.gov/pubmed/12373468) *Intens Care Med*. 2002;28:1434-1439.
24. Cohen J, Carlet J; [INTERSEPT: an international, multicenter, placebo-controlled trial of monoclonal antibody to human tumor necrosis factor-alpha in patients with sepsis. International Sepsis Trial Study Group.](http://www.ncbi.nlm.nih.gov/pubmed/8797612) *Crit Care Med*. 1996;24:1431-1440.
25. [Day NP](http://www.ncbi.nlm.nih.gov/pubmed?term="Day NP"%5BAuthor%5D), [Phu NH](http://www.ncbi.nlm.nih.gov/pubmed?term="Phu NH"%5BAuthor%5D), [Bethell DP](http://www.ncbi.nlm.nih.gov/pubmed?term="Bethell DP"%5BAuthor%5D), et al; The effects of dopamine and adrenaline infusions on acid-base balance and systemic haemodynamics in severe infection. *[Lancet.](http://www.ncbi.nlm.nih.gov/pubmed/8684198#%23)* 1996;348:219-223.
26. Day NP, Phu NH, Mai NT, et al; [Effects of dopamine and epinephrine infusions on renal hemodynamics in severe malaria and severe sepsis.](http://www.ncbi.nlm.nih.gov/pubmed/10834678) *Crit Care Med*. 2000;28:1353-1362.
27. [De Backer D](http://www.ncbi.nlm.nih.gov/pubmed?term="De Backer D"%5BAuthor%5D), [Berré J](http://www.ncbi.nlm.nih.gov/pubmed?term="Berré J"%5BAuthor%5D), [Zhang H](http://www.ncbi.nlm.nih.gov/pubmed?term="Zhang H"%5BAuthor%5D), [Kahn RJ](http://www.ncbi.nlm.nih.gov/pubmed?term="Kahn RJ"%5BAuthor%5D), [Vincent JL](http://www.ncbi.nlm.nih.gov/pubmed?term="Vincent JL"%5BAuthor%5D); Relationship between oxygen uptake and oxygen delivery in septic patients: effects of prostacyclin versus dobutamine. *[Crit Care Med.](http://www.ncbi.nlm.nih.gov/pubmed?term=de backer d 1993 prostaglandin " \l "%23)* 1993;21:1658-1664.
28. De Backer D, Creteur J, Silva E, Vincent JL; [Effects of dopamine, norepinephrine, and epinephrine on the splanchnic circulation in septic shock: which is best?](http://www.ncbi.nlm.nih.gov/pubmed/12794401) *Crit Care Med*. 2003;31:1659-1667.
29. [Dhainaut JF](http://www.ncbi.nlm.nih.gov/pubmed?term="Dhainaut JF"%5BAuthor%5D), [Tenaillon A](http://www.ncbi.nlm.nih.gov/pubmed?term="Tenaillon A"%5BAuthor%5D), [Hemmer M](http://www.ncbi.nlm.nih.gov/pubmed?term="Hemmer M"%5BAuthor%5D), et al ; Confirmatory platelet-activating factor receptor antagonist trial in patients with severe gram-negative bacterial sepsis: a phase III, randomized, double-blind, placebo-controlled, multicenter trial. BN 52021 Sepsis Investigator Group. *Crit Care Med.* 1998;26:1963-1971.
30. [Duranteau J](http://www.ncbi.nlm.nih.gov/pubmed?term="Duranteau J"%5BAuthor%5D), [Sitbon P](http://www.ncbi.nlm.nih.gov/pubmed?term="Sitbon P"%5BAuthor%5D), [Teboul JL](http://www.ncbi.nlm.nih.gov/pubmed?term="Teboul JL"%5BAuthor%5D), et al; Effects of epinephrine, norepinephrine, or the combination of norepinephrine and dobutamine on gastric mucosa in septic shock. *[Crit Care Med.](http://www.ncbi.nlm.nih.gov/pubmed/10362410#%23)* 1999;27:893-900.
31. Eisele B, Lamy M, Thijs LG, et al; [Antithrombin III in patients with severe sepsis. A randomized, placebo-controlled, double-blind multicenter trial plus a meta-analysis on all randomized, placebo-controlled, double-blind trials with antithrombin III in severe sepsis.](http://www.ncbi.nlm.nih.gov/pubmed/9722035) *Intensive Care Med*. 1998;24:663-672.
32. Fernandes CJ Jr, Akamine N, De Marco FV, De Souza JA, Lagudis S, Knobel E.[Red blood cell transfusion does not increase oxygen consumption in critically ill septic patients.](http://www.ncbi.nlm.nih.gov/pubmed/11737926) *Crit Care*. 2001;5:362-367.
33. [Fisher CJ Jr](http://www.ncbi.nlm.nih.gov/pubmed?term="Fisher CJ Jr"%5BAuthor%5D), [Dhainaut JF](http://www.ncbi.nlm.nih.gov/pubmed?term="Dhainaut JF"%5BAuthor%5D), [Opal SM](http://www.ncbi.nlm.nih.gov/pubmed?term="Opal SM"%5BAuthor%5D), et al; Recombinant human interleukin 1 receptor antagonist in the treatment of patients with sepsis syndrome. Results from a randomized, double-blind, placebo-controlled trial. Phase III rhIL-1ra Sepsis Syndrome Study Group. *[JAMA.](http://www.ncbi.nlm.nih.gov/pubmed?term=RHIL  1994 sepsis" \l "%23)* 1994;271:1836-1843.
34. Fisher CJ Jr, Agosti JM, Opal SM, et al; [Treatment of septic shock with the tumor necrosis factor receptor:Fc fusion protein. The Soluble TNF Receptor Sepsis Study Group.](http://www.ncbi.nlm.nih.gov/pubmed/8637514) *N Engl J Med*. 1996;334:1697-1702.
35. Forceville X, Laviolle B, Annane D, et al; [Effects of high doses of selenium, as sodium selenite, in septic shock: a placebo-controlled, randomized, double-blind, phase II study.](http://www.ncbi.nlm.nih.gov/pubmed/17617901) *Crit Care*. 2007;11:R73.
36. Fourrier F, Chopin C, Huart JJ, Runge I, Caron C, Goudemand J; [Double-blind, placebo-controlled trial of antithrombin III concentrates in septic shock with disseminated intravascular coagulation.](http://www.ncbi.nlm.nih.gov/pubmed/8365305) *Chest*. 1993;104:882-888.
37. Freebairn RC, Derrick J, Gomersall CD, Young RJ, Joynt GM; [Oxygen delivery, oxygen consumption, and gastric intramucosal pH are not improved by a computer-controlled, closed-loop, vecuronium infusion in severe sepsis and septic shock.](http://www.ncbi.nlm.nih.gov/pubmed/8989179) *Crit Care Med.* 1997;25:72-77.
38. Grover R, Zaccardelli D, Colice G, Guntupalli K, Watson D, Vincent JL; [An open-label dose escalation study of the nitric oxide synthase inhibitor, N(G)-methyl-L-arginine hydrochloride (546C88), in patients with septic shock. Glaxo Wellcome International Septic Shock Study Group.](http://www.ncbi.nlm.nih.gov/pubmed/10362413) *Crit Care Med*. 1999;27:913-922.
39. Hannemann L, Reinhart K, Meier-Hellmann A, Bredle DL; [Prostacyclin in septic shock.](http://www.ncbi.nlm.nih.gov/pubmed/8181344) *Chest*. 1994;105:1504-1510.
40. [Hannemann L](http://www.ncbi.nlm.nih.gov/pubmed?term="Hannemann L"%5BAuthor%5D), [Reinhart K](http://www.ncbi.nlm.nih.gov/pubmed?term="Reinhart K"%5BAuthor%5D), [Grenzer O](http://www.ncbi.nlm.nih.gov/pubmed?term="Grenzer O"%5BAuthor%5D), [Meier-Hellmann A](http://www.ncbi.nlm.nih.gov/pubmed?term="Meier-Hellmann A"%5BAuthor%5D), [Bredle DL](http://www.ncbi.nlm.nih.gov/pubmed?term="Bredle DL"%5BAuthor%5D); Comparison of dopamine to dobutamine and norepinephrine for oxygen delivery and uptake in septic shock. *[Crit Care Med.](http://www.ncbi.nlm.nih.gov/pubmed?term=dobutamin norepinephrin hannemann 1995" \l "%23)* 1995;23:1962-1970.
41. Hannemann L, Reinhart K, Meier-Hellmann A, Wallenfang G, Bredle DL; [Dopexamine hydrochloride in septic shock.](http://www.ncbi.nlm.nih.gov/pubmed/8617087) *Chest*. 1996;109:756-760.
42. [Hentrich M](http://www.ncbi.nlm.nih.gov/pubmed?term="Hentrich M"%5BAuthor%5D), [Fehnle K](http://www.ncbi.nlm.nih.gov/pubmed?term="Fehnle K"%5BAuthor%5D), [Ostermann H](http://www.ncbi.nlm.nih.gov/pubmed?term="Ostermann H"%5BAuthor%5D), et al; IgMA-enriched immunoglobulin in neutropenic patients with sepsis syndrome and septic shock: a randomized, controlled, multiple-center trial. *[Crit Care Med.](http://www.ncbi.nlm.nih.gov/pubmed?term=IgM hentrich" \l "%23)* 2006;34:1319-1325.
43. [Inthorn D](http://www.ncbi.nlm.nih.gov/pubmed?term="Inthorn D"%5BAuthor%5D), [Hoffmann JN](http://www.ncbi.nlm.nih.gov/pubmed?term="Hoffmann JN"%5BAuthor%5D), [Hartl WH](http://www.ncbi.nlm.nih.gov/pubmed?term="Hartl WH"%5BAuthor%5D), [Mühlbayer D](http://www.ncbi.nlm.nih.gov/pubmed?term="Mühlbayer D"%5BAuthor%5D), [Jochum M](http://www.ncbi.nlm.nih.gov/pubmed?term="Jochum M"%5BAuthor%5D); Antithrombin III supplementation in severe sepsis: beneficial effects on organ dysfunction. *[Shock.](http://www.ncbi.nlm.nih.gov/pubmed?term=inthorn 1997 antithrombin" \l "%23)* 1997;8:328-334.
44. [Jellema WT](http://www.ncbi.nlm.nih.gov/pubmed?term="Jellema WT"%5BAuthor%5D), [Groeneveld AB](http://www.ncbi.nlm.nih.gov/pubmed?term="Groeneveld AB"%5BAuthor%5D), [Wesseling KH](http://www.ncbi.nlm.nih.gov/pubmed?term="Wesseling KH"%5BAuthor%5D), [Thijs LG](http://www.ncbi.nlm.nih.gov/pubmed?term="Thijs LG"%5BAuthor%5D), [Westerhof N](http://www.ncbi.nlm.nih.gov/pubmed?term="Westerhof N"%5BAuthor%5D), [van Lieshout JJ](http://www.ncbi.nlm.nih.gov/pubmed?term="van Lieshout JJ"%5BAuthor%5D); Heterogeneity and prediction of hemodynamic responses to dobutamine in patients with septic shock. *[Crit Care Med.](http://www.ncbi.nlm.nih.gov/pubmed/16849997#%23)* 2006;34:2392-2398.
45. [Kaufmann I](http://www.ncbi.nlm.nih.gov/pubmed?term="Kaufmann I"%5BAuthor%5D), [Briegel J](http://www.ncbi.nlm.nih.gov/pubmed?term="Briegel J"%5BAuthor%5D), [Schliephake F](http://www.ncbi.nlm.nih.gov/pubmed?term="Schliephake F"%5BAuthor%5D), et al; Stress doses of hydrocortisone in septic shock: beneficial effects on opsonization-dependent neutrophil functions. *[Intens Care Med.](http://www.ncbi.nlm.nih.gov/pubmed?term=kaufmann steroid septic" \l "%23)* 2008;34:344-349.
46. Keh D, Boehnke T, Weber-Cartens S, Schulz C, et al; [Immunologic and hemodynamic effects of "low-dose" hydrocortisone in septic shock: a double-blind, randomized, placebo-controlled, crossover study.](http://www.ncbi.nlm.nih.gov/pubmed/12426230) *Am J Respir Crit Care Med*. 2003;167:512-520.
47. Kern H, Schröder T, Kaulfuss M, Martin M, Kox WJ, Spies CD; [Enoximone in contrast to dobutamine improves hepatosplanchnic function in fluid-optimized septic shock patients.](http://www.ncbi.nlm.nih.gov/pubmed/11505119) *Crit Care Med*. 2001;29:1519-1525.
48. Kirov MY, Evgenov OV, Evgenov NV, et al; [Infusion of methylene blue in human septic shock: a pilot, randomized, controlled study.](http://www.ncbi.nlm.nih.gov/pubmed/11588440) *Crit Care Med*. 2001;29:1860-1867.
49. Lauzier F, Lévy B, Lamarre P, Lesur O; [Vasopressin or norepinephrine in early hyperdynamic septic shock: a randomized clinical trial.](http://www.ncbi.nlm.nih.gov/pubmed/17019548) *Intensive Care Med*. 2006;32:1782-1789.
50. [Levy B](http://www.ncbi.nlm.nih.gov/pubmed?term="Levy B"%5BAuthor%5D), [Bollaert PE](http://www.ncbi.nlm.nih.gov/pubmed?term="Bollaert PE"%5BAuthor%5D), [Charpentier C](http://www.ncbi.nlm.nih.gov/pubmed?term="Charpentier C"%5BAuthor%5D), et al; Comparison of norepinephrine and dobutamine to epinephrine for hemodynamics, lactate metabolism, and gastric tonometric variables in septic shock: a prospective, randomized study. *[Intens Care Med.](http://www.ncbi.nlm.nih.gov/pubmed/9083230#%23)* 1997;23:282-287.
51. [Levy B](http://www.ncbi.nlm.nih.gov/pubmed?term="Levy B"%5BAuthor%5D), [Bollaert PE](http://www.ncbi.nlm.nih.gov/pubmed?term="Bollaert PE"%5BAuthor%5D), [Lucchelli JP](http://www.ncbi.nlm.nih.gov/pubmed?term="Lucchelli JP"%5BAuthor%5D), [Sadoune LO](http://www.ncbi.nlm.nih.gov/pubmed?term="Sadoune LO"%5BAuthor%5D), [Nace L](http://www.ncbi.nlm.nih.gov/pubmed?term="Nace L"%5BAuthor%5D), [Larcan A](http://www.ncbi.nlm.nih.gov/pubmed?term="Larcan A"%5BAuthor%5D); Dobutamine improves the adequacy of gastric mucosal perfusion in epinephrine-treated septic shock. *[Crit Care Med.](http://www.ncbi.nlm.nih.gov/pubmed/9377878#%23)* 1997;25:1649-1654.
52. [Levy B](http://www.ncbi.nlm.nih.gov/pubmed?term="Levy B"%5BAuthor%5D), [Nace L](http://www.ncbi.nlm.nih.gov/pubmed?term="Nace L"%5BAuthor%5D), [Bollaert PE](http://www.ncbi.nlm.nih.gov/pubmed?term="Bollaert PE"%5BAuthor%5D), [Dousset B](http://www.ncbi.nlm.nih.gov/pubmed?term="Dousset B"%5BAuthor%5D), [Mallie JP](http://www.ncbi.nlm.nih.gov/pubmed?term="Mallie JP"%5BAuthor%5D), [Larcan A](http://www.ncbi.nlm.nih.gov/pubmed?term="Larcan A"%5BAuthor%5D); Comparison of systemic and regional effects of dobutamine and dopexamine in norepinephrine-treated septic shock. *[Intens Care Med.](http://www.ncbi.nlm.nih.gov/pubmed?term=dobutamin dopexamin norepinephrin levy  septic" \l "%23)* 1999;25:942-948.
53. Loisa P, Parviainen I, Tenhunen J, Hovilehto S, Ruokonen E; [Effect of mode of hydrocortisone administration on glycemic control in patients with septic shock: a prospective randomized trial.](http://www.ncbi.nlm.nih.gov/pubmed/17306016) Crit Care. 2007;11:R21.
54. López A, Lorente JA, Steingrub J, et al; [Multiple-center, randomized, placebo-controlled, double-blind study of the nitric oxide synthase inhibitor 546C88: effect on survival in patients with septic shock.](http://www.ncbi.nlm.nih.gov/pubmed/14707556) *Crit Care Med*. 2004;32:21-30.
55. [Lorente JA](http://www.ncbi.nlm.nih.gov/pubmed?term="Lorente JA"%5BAuthor%5D), [Landín L](http://www.ncbi.nlm.nih.gov/pubmed?term="Landín L"%5BAuthor%5D), [De Pablo R](http://www.ncbi.nlm.nih.gov/pubmed?term="De Pablo R"%5BAuthor%5D), [Renes E](http://www.ncbi.nlm.nih.gov/pubmed?term="Renes E"%5BAuthor%5D), [Rodríguez-Díaz R](http://www.ncbi.nlm.nih.gov/pubmed?term="Rodríguez-Díaz R"%5BAuthor%5D), [Liste D](http://www.ncbi.nlm.nih.gov/pubmed?term="Liste D"%5BAuthor%5D) ; Effects of blood transfusion on oxygen transport variables in severe sepsis. *[Crit Care Med.](http://www.ncbi.nlm.nih.gov/pubmed/8370294#%23)* 1993;21:1312-1318.
56. [Malay MB](http://www.ncbi.nlm.nih.gov/pubmed?term="Malay MB"%5BAuthor%5D), [Ashton RC Jr](http://www.ncbi.nlm.nih.gov/pubmed?term="Ashton RC Jr"%5BAuthor%5D), [Landry DW](http://www.ncbi.nlm.nih.gov/pubmed?term="Landry DW"%5BAuthor%5D), [Townsend RN](http://www.ncbi.nlm.nih.gov/pubmed?term="Townsend RN"%5BAuthor%5D); Low-dose vasopressin in the treatment of vasodilatory septic shock. *[J Trauma.](http://www.ncbi.nlm.nih.gov/pubmed?term=malay vasopressin 1999 sepsis" \l "%23)* 1999;47:699-703.
57. Marik PE, Mohedin M; [The contrasting effects of dopamine and norepinephrine on systemic and splanchnic oxygen utilization in hyperdynamic sepsis.](http://www.ncbi.nlm.nih.gov/pubmed/7933396) *JAMA*. 1994;272:1354-1357.
58. Martin C, Papazian L, Perrin G, Saux P, Gouin F ; [Norepinephrine or dopamine for the treatment of hyperdynamic septic shock?](http://www.ncbi.nlm.nih.gov/pubmed/8404107) *Chest*. 1993;103:1826-1831.
59. [Martin C](http://www.ncbi.nlm.nih.gov/pubmed?term="Martin C"%5BAuthor%5D), [Viviand X](http://www.ncbi.nlm.nih.gov/pubmed?term="Viviand X"%5BAuthor%5D), [Arnaud S](http://www.ncbi.nlm.nih.gov/pubmed?term="Arnaud S"%5BAuthor%5D), [Vialet R](http://www.ncbi.nlm.nih.gov/pubmed?term="Vialet R"%5BAuthor%5D), [Rougnon T](http://www.ncbi.nlm.nih.gov/pubmed?term="Rougnon T"%5BAuthor%5D); Effects of norepinephrine plus dobutamine or norepinephrine alone on left ventricular performance of septic shock patients. *[Crit Care Med.](http://www.ncbi.nlm.nih.gov/pubmed?term=dobutamin shock septic 1999 martin " \l "%23)* 1999;27:1708-1713.
60. Meier-Hellmann A, Bredle DL, Specht M, Hannemann L, Reinhart K. [Dopexamine increases splanchnic blood flow but decreases gastric mucosal pH in severe septic patients treated with dobutamine.](http://www.ncbi.nlm.nih.gov/pubmed/10548200) *Crit Care Med*. 1999;27:2166-2171.
61. [Moran JL](http://www.ncbi.nlm.nih.gov/pubmed?term="Moran JL"%5BAuthor%5D), [O'Fathartaigh MS](http://www.ncbi.nlm.nih.gov/pubmed?term="O'Fathartaigh MS"%5BAuthor%5D), [Peisach AR](http://www.ncbi.nlm.nih.gov/pubmed?term="Peisach AR"%5BAuthor%5D), [Chapman MJ](http://www.ncbi.nlm.nih.gov/pubmed?term="Chapman MJ"%5BAuthor%5D), [Leppard P](http://www.ncbi.nlm.nih.gov/pubmed?term="Leppard P"%5BAuthor%5D); Epinephrine as an inotropic agent in septic shock: a dose-profile analysis. *[Crit Care Med.](http://www.ncbi.nlm.nih.gov/pubmed/8420733#%23)* 1993;21:70-77.
62. Morelli A, Ricci Z, Bellomo R, et al; [Prophylactic fenoldopam for renal protection in sepsis: a randomized, double-blind, placebo-controlled pilot trial.](http://www.ncbi.nlm.nih.gov/pubmed/16276165) *Crit Care Med*. 2005;33:2451-2456.
63. Morelli A, Lange M, Ertmer C, et al; [Glibenclamide dose response in patients with septic shock: effects on norepinephrine requirements, cardiopulmonary performance, and global oxygen transport.](http://www.ncbi.nlm.nih.gov/pubmed/17589379) *Shock*. 2007;28:530-535.
64. Morelli A, Ertmer C, Lange M, et al; [Effects of short-term simultaneous infusion of dobutamine and terlipressin in patients with septic shock: the DOBUPRESS study.](http://www.ncbi.nlm.nih.gov/pubmed/18308741) *Br J Anaesth*. 2008;100:494-503.
65. Morelli A, Ertmer C, Rehberg S, et al; [Phenylephrine versus norepinephrine for initial hemodynamic support of patients with septic shock: a randomized, controlled trial.](http://www.ncbi.nlm.nih.gov/pubmed/19017409) *Crit Care*. 2008;12:R143.
66. Mussack T, Briegel J, Schelling G, Jochum M; [Hemofiltration does not influence early S-100B serum levels in septic shock patients receiving stress doses of hydrocortisone or placebo.](http://www.ncbi.nlm.nih.gov/pubmed/15817428) *Eur J Med Res*. 2005;10:81-87.
67. Nakamura T, Kawagoe Y, Matsuda T, Koide H ; [Effect of polymyxin B-immobilized fiber on bone resorption in patients with sepsis.](http://www.ncbi.nlm.nih.gov/pubmed/15197430) *Intens Care Med*. 2004;30:1838-1841.
68. [Natalini G](http://www.ncbi.nlm.nih.gov/pubmed?term="Natalini G"%5BAuthor%5D), [Schivalocchi V](http://www.ncbi.nlm.nih.gov/pubmed?term="Schivalocchi V"%5BAuthor%5D), [Rosano A](http://www.ncbi.nlm.nih.gov/pubmed?term="Rosano A"%5BAuthor%5D), [Taranto M](http://www.ncbi.nlm.nih.gov/pubmed?term="Taranto M"%5BAuthor%5D), [Pletti C](http://www.ncbi.nlm.nih.gov/pubmed?term="Pletti C"%5BAuthor%5D), [Bernardini A](http://www.ncbi.nlm.nih.gov/pubmed?term="Bernardini A"%5BAuthor%5D); Norepinephrine and metaraminol in septic shock: a comparison of the hemodynamic effects. *Intens Care Med*. 2005;31:634-637.
69. [Opal SM](http://www.ncbi.nlm.nih.gov/pubmed?term="Opal SM"%5BAuthor%5D), [Fisher CJ Jr](http://www.ncbi.nlm.nih.gov/pubmed?term="Fisher CJ Jr"%5BAuthor%5D), [Dhainaut JF](http://www.ncbi.nlm.nih.gov/pubmed?term="Dhainaut JF"%5BAuthor%5D), et al; Confirmatory interleukin-1 receptor antagonist trial in severe sepsis: a phase III, randomized, double-blind, placebo-controlled, multicenter trial. The Interleukin-1 Receptor Antagonist Sepsis Investigator Group. *[Crit Care Med.](http://www.ncbi.nlm.nih.gov/pubmed?term=randomized opal s 1997 interleukin" \l "%23)* 1997;25:1115-1124.
70. Oppert M, Schindler R, Husung C, et al; [Low-dose hydrocortisone improves shock reversal and reduces cytokine levels in early hyperdynamic septic shock.](http://www.ncbi.nlm.nih.gov/pubmed/16276166) *Crit Care Med*. 2005;33:2457-2464.
71. Patel BM, Chittock DR, Russell JA, Walley KR; [Beneficial effects of short-term vasopressin infusion during severe septic shock.](http://www.ncbi.nlm.nih.gov/pubmed/11873030) *Anesthesiology*. 2002;96:576-582.
72. [Peake SL](http://www.ncbi.nlm.nih.gov/pubmed?term="Peake SL"%5BAuthor%5D), [Moran JL](http://www.ncbi.nlm.nih.gov/pubmed?term="Moran JL"%5BAuthor%5D), [Leppard PI](http://www.ncbi.nlm.nih.gov/pubmed?term="Leppard PI"%5BAuthor%5D); N-acetyl-L-cysteine depresses cardiac performance in patients with septic shock. *[Crit Care Med.](http://www.ncbi.nlm.nih.gov/pubmed?term=peake s acetyl cystein septic" \l "%23)* 1996;24:1302-1310.
73. Pittet D, Harbarth S, Suter PM, et al; [Impact of immunomodulating therapy on morbidity in patients with severe sepsis.](http://www.ncbi.nlm.nih.gov/pubmed/10471608) *Am J Respir Crit Care Med*. 1999;160:852-857.
74. [Rank N](http://www.ncbi.nlm.nih.gov/pubmed?term="Rank N"%5BAuthor%5D), [Michel C](http://www.ncbi.nlm.nih.gov/pubmed?term="Michel C"%5BAuthor%5D), [Haertel C](http://www.ncbi.nlm.nih.gov/pubmed?term="Haertel C"%5BAuthor%5D), [et](http://www.ncbi.nlm.nih.gov/pubmed?term="Lenhart A"%5BAuthor%5D) al; N-acetylcysteine increases liver blood flow and improves liver function in septic shock patients: results of a prospective, randomized, double-blind study. *[Crit Care Med.](http://www.ncbi.nlm.nih.gov/pubmed?term=acetylcystein septic shock rank" \l "%23)* 2000;28:3799-3807.
75. Redl-Wenzl EM, Armbruster C, Edelmann G, et al; [The effects of norepinephrine on hemodynamics and renal function in severe septic shock states.](http://www.ncbi.nlm.nih.gov/pubmed/8315122) *Intens Care Med*. 1993;19:151-154.
76. [Reinelt H](http://www.ncbi.nlm.nih.gov/pubmed?term="Reinelt H"%5BAuthor%5D), [Radermacher P](http://www.ncbi.nlm.nih.gov/pubmed?term="Radermacher P"%5BAuthor%5D), [Kiefer P](http://www.ncbi.nlm.nih.gov/pubmed?term="Kiefer P"%5BAuthor%5D), et al; Impact of exogenous beta-adrenergic receptor stimulation on hepatosplanchnic oxygen kinetics and metabolic activity in septic shock. *[Crit Care Med.](http://www.ncbi.nlm.nih.gov/pubmed?term=phenylephrin reinelt" \l "%23)* 1999;27:325-331.
77. Reinhart K, Meier-Hellmann A, Beale R, et al; EASy-Study Group. [Open randomized phase II trial of an extracorporeal endotoxin adsorber in suspected Gram-negative sepsis.](http://www.ncbi.nlm.nih.gov/pubmed/15286541) *Crit Care Med*. 2004;32:1662-1668.
78. [Rhodes A](http://www.ncbi.nlm.nih.gov/pubmed?term="Rhodes A"%5BAuthor%5D), [Lamb FJ](http://www.ncbi.nlm.nih.gov/pubmed?term="Lamb FJ"%5BAuthor%5D), [Malagon I](http://www.ncbi.nlm.nih.gov/pubmed?term="Malagon I"%5BAuthor%5D), [Newman PJ](http://www.ncbi.nlm.nih.gov/pubmed?term="Newman PJ"%5BAuthor%5D), [Grounds RM](http://www.ncbi.nlm.nih.gov/pubmed?term="Grounds RM"%5BAuthor%5D), [Bennett ED](http://www.ncbi.nlm.nih.gov/pubmed?term="Bennett ED"%5BAuthor%5D); A prospective study of the use of a dobutamine stress test to identify outcome in patients with sepsis, severe sepsis, or septic shock. *[Crit Care Med.](http://www.ncbi.nlm.nih.gov/pubmed?term=1999 rhodes  septic" \l "%23)* 1999;27:2361-2366.
79. [Rivers E](http://www.ncbi.nlm.nih.gov/pubmed?term="Rivers E"%5BAuthor%5D), [Nguyen B](http://www.ncbi.nlm.nih.gov/pubmed?term="Nguyen B"%5BAuthor%5D), [Havstad S](http://www.ncbi.nlm.nih.gov/pubmed?term="Havstad S"%5BAuthor%5D), et al; [Early Goal-Directed Therapy Collaborative Group](http://www.ncbi.nlm.nih.gov/pubmed?term="Early Goal-Directed Therapy Collaborative Group"%5BCorporate Author%5D). Early goal-directed therapy in the treatment of severe sepsis and septic shock. *[N Engl J Med.](http://www.ncbi.nlm.nih.gov/pubmed?term=rivers e early goal n engl j med  sepsis 2001" \l "%23)* 2001;345:1368-1377.
80. Russell JA, Walley KR, Singer J, et al; [Vasopressin versus norepinephrine infusion in patients with septic shock.](http://www.ncbi.nlm.nih.gov/pubmed/18305265) *N Engl J Med*. 2008;358:877-887.
81. Sakr Y, Reinhardt K, Vincent JL, et al. Does dopamine administration in shock influence outcome? Results of the Sepsis Occurrence in Acutely Ill Patients (SOAP) Study. *Crit Care Med*. 2006;34:589–597.
82. Schaffartzik W, Sanft C, Schaefer JH, Spies C; [Different dosages of dobutamine in septic shock patients: determining oxygen consumption with a metabolic monitor integrated in a ventilator.](http://www.ncbi.nlm.nih.gov/pubmed/11271080) *Intens Care Med*. 2000;26:1740-1746.
83. Schmoelz M, Schelling G, Dunker M, Irlbeck M; [Comparison of systemic and renal effects of dopexamine and dopamine in norepinephrine-treated septic shock.](http://www.ncbi.nlm.nih.gov/pubmed/16616656) *J Cardiothorac Vasc Anesth*. 2006;20:173-178.
84. Seguin P, Laviolle B, Guinet P, Morel I, Mallédant Y, Bellissant E ; [Dopexamine and norepinephrine versus epinephrine on gastric perfusion in patients with septic shock: a randomized study [NCT00134212].](http://www.ncbi.nlm.nih.gov/pubmed/16507156) *Crit Care*. 2006;10:R32.
85. Seguin P, Bellissant E, Le Tulzo Y, et al; [Effects of epinephrine compared with the combination of dobutamine and norepinephrine on gastric perfusion in septic shock.](http://www.ncbi.nlm.nih.gov/pubmed/12011824) *Clin Pharmacol Ther.* 2002;71:381-388.
86. Spies CD, Reinhart K, Witt I, et al; [Influence of N-acetylcysteine on indirect indicators of tissue oxygenation in septic shock patients: results from a prospective, randomized, double-blind study.](http://www.ncbi.nlm.nih.gov/pubmed/7956276) *Crit Care Med*. 1994;22:1738-1746.
87. Staubach KH, Schröder J, Stüber F, Gehrke K, Traumann E, Zabel P; [Effect of pentoxifylline in severe sepsis: results of a randomized, double-blind, placebo-controlled study.](http://www.ncbi.nlm.nih.gov/pubmed/9438767) *Arch Surg.* 1998;133:94-100.
88. Stephens DP, Thomas JH, Higgins A, et al; [Randomized, double-blind, placebo-controlled trial of granulocyte colony-stimulating factor in patients with septic shock.](http://www.ncbi.nlm.nih.gov/pubmed/18216600) *Crit Care Med*. 2008;36:448-454.
89. Tuchschmidt J, Fried J, Astiz M, Rackow E; [Elevation of cardiac output and oxygen delivery improves outcome in septic shock.](http://www.ncbi.nlm.nih.gov/pubmed/1623756) *Chest*. 1992;102:216-220.
90. [van Haren FM](http://www.ncbi.nlm.nih.gov/pubmed?term="van Haren FM"%5BAuthor%5D), [Rozendaal FW](http://www.ncbi.nlm.nih.gov/pubmed?term="Rozendaal FW"%5BAuthor%5D), [van der Hoeven JG](http://www.ncbi.nlm.nih.gov/pubmed?term="van der Hoeven JG"%5BAuthor%5D); The effect of vasopressin on gastric perfusion in catecholamine-dependent patients in septic shock. *[Chest.](http://www.ncbi.nlm.nih.gov/pubmed?term=The Effect of Vasopressin on Gastric Perfusion in Catecholamine-Dependent Patients in Septic Shock*" \l "%23)* 2003;124:2256-2260.
91. Vincent JL, Spapen H, Bakker J, Webster NR, Curtis L; [Phase II multicenter clinical study of the platelet-activating factor receptor antagonist BB-882 in the treatment of sepsis.](http://www.ncbi.nlm.nih.gov/pubmed/10752807) *Crit Care Med*. 2000;28:638-642.
92. Warren BL, Eid A, Singer P, et al; [High-dose antithrombin III in severe sepsis: a randomized controlled trial.](http://www.ncbi.nlm.nih.gov/pubmed/11597289) *JAMA*. 2001;286:1869-1878.
93. [Warrillow S](http://www.ncbi.nlm.nih.gov/pubmed?term="Warrillow S"%5BAuthor%5D), [Egi M](http://www.ncbi.nlm.nih.gov/pubmed?term="Egi M"%5BAuthor%5D), [Bellomo R](http://www.ncbi.nlm.nih.gov/pubmed?term="Bellomo R"%5BAuthor%5D); Randomized, double-blind, placebo-controlled crossover pilot study of a potassium channel blocker in patients with septic shock. *[Crit Care Med.](http://www.ncbi.nlm.nih.gov/pubmed?term=glibenclamid palcebo  sepsis" \l "%23)* 2006;34:980-985.
94. Yu M, Levy MM, Smith P, Takiguchi SA, Miyasaki A, Myers SA; [Effect of maximizing oxygen delivery on morbidity and mortality rates in critically ill patients: a prospective, randomized, controlled study.](http://www.ncbi.nlm.nih.gov/pubmed/8504649) *Crit Care Med*. 1993;21:830-838.
95. Zeni F, Pain P, Vindimian M, et al; [Effects of pentoxifylline on circulating cytokine concentrations and hemodynamics in patients with septic shock: results from a double-blind, randomized, placebo-controlled study.](http://www.ncbi.nlm.nih.gov/pubmed/8605790) *Crit Care Med*. 1996;24:207-214.
96. Zhou SX, Qiu HB, Huang YZ, Yang Y, Zheng RQ; [Effects of norepinephrine, epinephrine, and norepinephrine-dobutamine on systemic and gastric mucosal oxygenation in septic shock.](http://www.ncbi.nlm.nih.gov/pubmed/12100762) *Acta Pharmacol Sin*. 2002;23:654-658.
