# Supplementary material for: Are clinical trials dealing with severe infection fitting routine practices? Insights from a large registry
Source: Crit Care. 2013 May 24;17(3):R89. doi: 10.1186/cc12734 (PMC3706971; doi:10.1186/cc12734)
Supplement: Additional file 3 — a figure showing the time trends of severe immune suppression in septic patients from the Cub-Rea Database (1993 to 2008). Severe immune suppression combined transplant, neutropenia and AIDS. Year 1992 was removed from the graph because of nonrepresentative values related to the small sample. [file cc12734-S3.DOC]

Additional file 3, Figure S3.


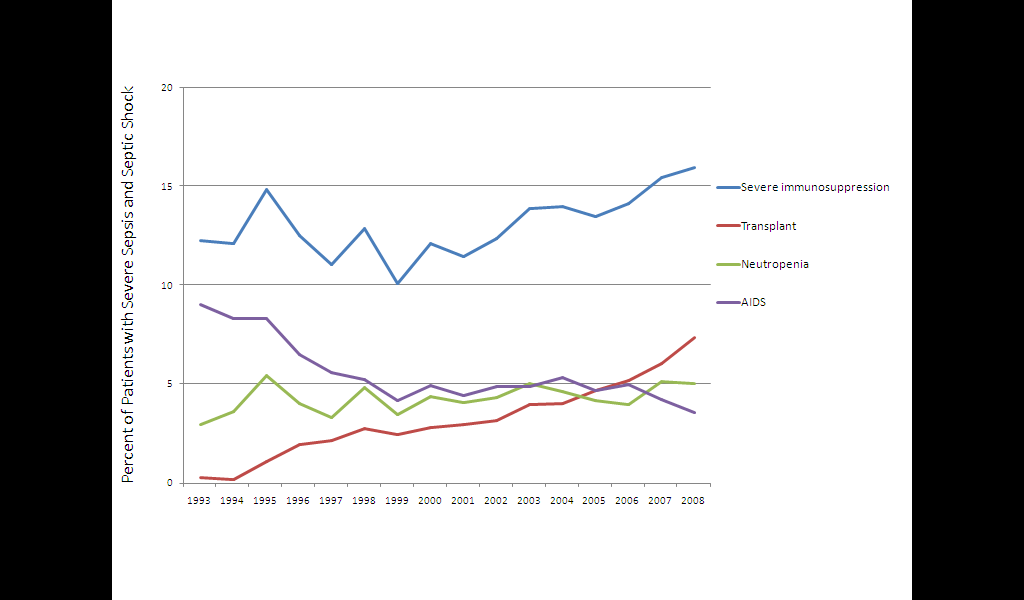
Severe Immune Suppression combined Transplant, Neutropenia and AIDS. Year 1992 was removed from the graph because of non representative values related to the small sample.
