# Supplementary material for: Are clinical trials dealing with severe infection fitting routine practices? Insights from a large registry
Source: Crit Care. 2013 May 24;17(3):R89. doi: 10.1186/cc12734 (PMC3706971; doi:10.1186/cc12734)
Supplement: Additional file 4 — a list presenting the classification of non-inclusion criteria according to their justification [17]. [file cc12734-S4.DOC]

**Additional file 4, Table S4. Classification of non-inclusion criteria according to their justification [17].**

**Strongly Justified Reasons for Excluding Individuals From a Randomized Controlled Trial**

Individual or substitute decision maker is unable to grant informed consent

Intervention or placebo would likely be harmful

Unacceptable risk of known adverse reaction to intervention

Unacceptable risk of assignment to placebo or withholding of intervention

Intervention would likely be ineffective

Individual not likely to have the condition of interest

Individual not at risk for outcome

Individual has type of disease that is likely not to respond to treatment

Effect of intervention will be difficult to interpret

Individual has a cointervention that will likely confound the treatment effect

Individual has an independent condition with signs and symptoms similar to

the condition of interest that will make the treatment effect difficult to assess

(eg, allergic rhinitis and upper respiratory tract infection)

**Poorly Justified Reasons for Excluding Individuals From a Trial**

Is not a strongly justifiable reason as described above

All of the following are true:

The exclusion is based on 1 or more of the following factors:

Age

Sex

Sex-specific conditions such as menstruation, pregnancy, or lactation

Racial, ethnic, or religious background

Spoken or written language ability

Educational background

Socioeconomic status

Cognitive ability or IQ

Physical ability or disability

Chronic health condition

The condition under investigation and/or the intervention is not specific to the

factors described above

The factors described above have no direct bearing on the condition, intervention,

or results

**Potentially Justified Reasons for Excluding Individuals From a Trial**

Is neither a strongly justified reason nor a poorly justified reason as described above

Individual may not adhere to intervention

Individual may not complete follow-up
