# Supplementary material for: Are clinical trials dealing with severe infection fitting routine practices? Insights from a large registry
Source: Crit Care. 2013 May 24;17(3):R89. doi: 10.1186/cc12734 (PMC3706971; doi:10.1186/cc12734)
Supplement: Additional file 5 — a table presenting the frequency of poorly justified reasons among main non-inclusion criteria across studies' categories [17]. Results are expressed as number of poorly justified reason for non-inclusion/number of studies with each non-inclusion criterion (%). [file cc12734-S5.DOC]

**Additional file 5, Table S5. Frequency of poorly justified reasons among main non-inclusion criteria across studies’ categories [17]. Results are expressed as number of poorly justified reason for non inclusion / number of studies with each non-inclusion criterion (%).**

| *Non inclusion criteria* | *Vasopressor* | *Fluid loading* | *Steroids* | *Modulation of immunity* | *Modulation of coagulation* | | *Miscellaneous* |
| --- | --- | --- | --- | --- | --- | --- | --- |
| Pregnancy  Age<18y  Age>75y  Congestive heart failure  Cancer  Gastrointestinal & liver  Use of steroids  HIV infection  Solid organ graft  Coagulation abnormalities | 16/16 (100%)  7/7 (100%)  1/1 (100%)  13/13 (100%)  7/7 (100%)  6/6 (100%)  0  0  0  0 | 2/2 (100%)  6/6 (100%)  1/1 (100%)  2/2 (100%)  2/2 (100%)  2/2 (100%)  0  1/1 (100%)  1/1 (100%)  1/1 (100%) | 4/4 (100%)  5/5 (100%)  1/1 (100%)  3/3 (100%)  3/3 (100%)  2/2 (100%)  0/7 (0%)  3/3 (100%)  0/4 (0%)  2/2 (100%) | 8/15 (53%)  13/13 (100%)  1/1 (100%)  12/12 (100%)  11/11 (100%)  6/6 (100%)  9/11 (81%)  9/9 (100%)  5/7 (71%)  5/5 (100%) | 0/6 (0%)  7/7 (100%)  0  2/2 (100%)  6/6 (100%)  0/3 (0%)  0  1/1 (100%)  2/2 (100%)  0/6 (0%) | 4 (100%)  1/1 (100%)  0  1/1 (100%)  2/2 (100%)  0/1 (0%)  0  0  0  0 | |
