# Supplementary material for: Are clinical trials dealing with severe infection fitting routine practices? Insights from a large registry
Source: Crit Care. 2013 May 24;17(3):R89. doi: 10.1186/cc12734 (PMC3706971; doi:10.1186/cc12734)
Supplement: Additional file 6 — a table presenting the non-inclusion criteria in studies published in high impact factor journals. [file cc12734-S6.DOC]

**Additional file 6, Table S6**. Non inclusion criteria in studies published in high impact factors journals.

| *Author, year* | Pregnancy | Age<18y | Age>75y | Congestive heart failure | Cancer | Gastrointestinal & liver disorders | Use of steroids | HIV infection | Solid organ graft | Coagulation abnormalities |
| --- | --- | --- | --- | --- | --- | --- | --- | --- | --- | --- |
| Abraham, 1998  Abraham, 2003  Abraham, 2005  Annane, 2002  Annane, 2007  Bernard, 2001  Brunkhorst, 2008  Fisher, 1994  Fisher, 1996  Rivers, 2001  Russel, 2008  Warren, 2001 | Included  Non included  Included  Non included  Non included  Non included  Non included  Non included  Non included  Non included  Non included  Non included | Non included  Non included  Included  Non included  Non included  Included  Included  Non included  Non included  Non included  Included  Included | Included  Included  Included  Included  Included  Included  Included  Included  Included  Included  Included  Included | Included  Non included  Included  Non included  Non included  Included  Included  Non included  Non included  Non included  Non included  Included | Non included  Included  Non included  Non included  Non included  Non included  Included  Non included  Non included  Non included  Non included  Non included | Included  Non included  Included  Non included  Included  Included  Included  Non included  Included  Non included  Included  Non included | Included  Included  Included  Included  Included  Included  Included  Included  Non included  Included  Included  Included | Non included  Included  Included  Non included  Non included  Included  Included  Included  Non included  Non included  Included  Non included | Included  Non included  Included  Non included  Included  Included  Included  Non included  Non included  Included  Included  Included | Included  Non included  Non included  Non included  Included  Non included  Included  Included  Included  Non included  Included  Non included |
